# Characteristics and Physical Properties of Indonesian Stingless Bee (*Geniotrigona thoracica*) Propolis‐Loaded Chitosan Oligosaccharide Nanoparticles as a Root Canal Nanosealer With Bioceramic Sealers

**DOI:** 10.1155/ijod/5895286

**Published:** 2026-08-09

**Authors:** Trimurni Abidin, Muhammad Rizki, Widi Prasetia, Nandi Sukri, Wandania Farahanny, Nevi Yanti, Harry Agusnar, Ronny Lesmana, Felix Zulhendri

**Affiliations:** ^1^ Department of Conservative Dentistry, Faculty of Dentistry, Universitas Sumatera Utara, Medan, Indonesia, usu.ac.id; ^2^ Department of Food Technology, Faculty of Agricultural Industrial Technology, Universitas Padjadjaran, Kabupaten Sumedang, Jawa Barat, Indonesia, unpad.ac.id; ^3^ Department of Chemistry, Faculty of Mathematics and Science, Universitas Sumatera Utara, Medan, Indonesia, usu.ac.id; ^4^ Physiology Division, Department of Biomedical Sciences, Faculty of Medicine, Universitas Padjadjaran, Bandung, Indonesia, unpad.ac.id; ^5^ Department of Pharmacology and Clinical Pharmacy, Faculty of Pharmacy, Universitas Padjadjaran, Kabupaten Sumedang 45363, Jawa Barat, Indonesia, unpad.ac.id; ^6^ Kebun Efi, Kabupaten Karo 22171, Sumatera Utara, Indonesia

**Keywords:** bioceramics, chitosan-propolis, endodontic sealer, mechanical properties, nanoparticles

## Abstract

**Objectives:**

This study aimed to assess the suitability of a novel natural‐based endodontic sealer, nano‐chitosan‐propolis, for root canal applications by comparing its physicochemical and structural properties with a commercial bioceramic sealer (CeraSeal).

**Methods:**

The physicochemical and structural characteristics of nano‐chitosan oligosaccharide‐propolis (nCOS‐P) and CeraSeal were evaluated using X‐ray diffraction (XRD) to identify crystalline phases and scanning electron microscopy (SEM), equipped with energy‐dispersive X‐ray spectroscopy (EDS) (SEM‐EDS) for surface morphology and elemental composition. Setting time was measured following the ASTM C191‐08 standard under room conditions (23 ± 1^∘^C). The flowability and film thickness were tested following ISO 6876/2012 standards to meet standard criteria as an ideal endodontic sealer.

**Results:**

XRD analysis revealed that nCOS‐P exhibited crystalline phases of calcium, zirconium, zirconium dioxide, and silicon dioxide, whereas CeraSeal contained zirconium silicide, indicating compositional differences. SEM‐EDS showed that nCOS‐P had a compact, nonuniform particle distribution and elemental composition consistent with chitosan and propolis, contributing to enhanced viscosity and bioactivity. nCOS‐P demonstrated a significantly shorter setting time (1320 min) compared to CeraSeal (3360 min) (*p*  < 0.05). Flowability of nCOS‐P was slightly higher (19.05 ± 0.36 mm) compared with CeraSeal (18.15 ± 0.26 mm) (*p* = 0.021). The nCOS‐P had a film thickness of 180 ± 1.45 µm, whereas CeraSeal had a film thickness of 260 ± 0.11 µm. However, both materials exceeded the ISO 6876 limit ( ≤50 µm) under the present test conditions.

**Conclusions:**

nCOS‐P demonstrated a shorter ASTM‐based setting time and acceptable flowability. Still, neither material met the ISO film thickness limit under the present conditions. Despite exceeding ISO limits for film thickness, its unique composition offers potential clinical advantages. Further in vivo studies are recommended to confirm biological performance and clinical efficacy.

## 1. Introduction

The root canal filling process primarily aims to three‐dimensionally seal the entire pulp chamber to prevent microbial invasion, fluid leakage, and accumulation, which can lead to reinfection [1, 2]. However, successful root canal filling is often challenging due to the complexity of the root canal’s anatomical structure. The complexity of the root canal system and dentinal tubule architecture limits the complete adaptation and penetration of obturation materials [3, 4].

In this context, endodontic sealers are essential for filling the space between the dentin wall and core materials such as gutta‐percha. Selecting a sealer with appropriate characteristics is crucial to ensure good sealing ability, prevent microleakage, and improve fracture resistance [5–8]. The ideal characteristics of sealers include effective penetration into dentinal tubules, optimal density, good flowability, low solubility, and adequate radiopacity. In addition, antimicrobial properties, biocompatibility, and the capacity to support biological tissue regeneration are also important factors [9, 10]. Technological developments, including nanomaterials, have provided partial solutions, but shortcomings remain in the overall effectiveness of the materials used [11, 12].

Among currently available materials, calcium silicate (CaSi)‐based bioceramic sealers have attracted considerable interest because of their biointeractive properties [13]. In phosphate‐containing environments, CaSi hydration releases calcium and hydroxyl ions, creating an alkaline microenvironment while forming a hydrated silica‐rich surface that promotes the nucleation of amorphous calcium phosphate and its subsequent maturation into apatite‐like deposits, thereby enhancing mineralization at the dentin–sealer interface [13]. Clinically, premixed CaSi sealers are valued for their ease of application, favorable flow characteristics, ion release‐mediated bioactivity, and ability to stimulate regenerative responses in mesenchymal stem cells [14]. However, they remain moisture‐dependent and may exhibit delayed or incomplete setting under low‐humidity conditions [15]. Radiopacity is also an essential property for evaluating obturation quality and detecting voids, as specified by ISO 6876, with modern sealers commonly incorporating zirconium oxide, tantalum oxide, or bismuth oxide as radiopacifiers [16].

The incorporation of natural biopolymers and bioactive compounds into CaSi sealers offers a promising approach to improve their biological performance and sustainability. Propolis possesses antibacterial, anti‐inflammatory, antioxidant, and autophagy‐modulating activities that may enhance sealer bioactivity [17–19]. Indonesian stingless bee (*Geniotrigona thoracica*) propolis from North Sumatra has demonstrated wound‐healing and anti‐inflammatory effects in animal models and is rich in triterpenoids such as cycloartenol, amyrenone, and mangiferolic acid [20–23]. Chitosan oligosaccharide also exhibits antibacterial, antioxidant, and anti‐inflammatory properties, and its combination with propolis nanoparticles has been reported to improve dentinal tubule penetration, reduce absorption values, and enhance adhesion to the root canal wall, highlighting its potential to optimize the physical and biological characteristics of endodontic sealers [2, 24].

Previous studies have explored the use of natural material‐based nanoparticles in endodontic sealers to enhance antibacterial effectiveness. However, there remains a lack of material development based on the combination of propolis and chitosan. This combination is expected to produce an innovative root canal filling material that not only improves the physical properties of the sealer but also enhances sealing ability and supports tissue regeneration in the periapical region [2, 25].

Therefore, this study aims to develop a novel sealer, nano‐chitosan oligosaccharide‐propolis (nCOS‐P), which is expected to meet the ideal physical and biological characteristics required for root canal filling materials. The novelty of this research lies in synthesizing natural ingredients: propolis and water‐soluble chitosan oligosaccharide, that will be evaluated for their physical properties. In this study, the term “nanosealer” refers specifically to the incorporation of chitosan‐propolis nanoparticles within the sealer matrix rather than implying that the entire sealer particle population is nanoscale.

## 2. Materials and Method

### 2.1. Research Design

This research is a laboratory‐based experimental study with a comparative test design, conducted to compare the physical properties of nano‐chitosan‐propolis sealers with those of a commercial bioceramic sealer (CeraSeal). The samples consisted of nCOS‐P sealers synthesized from a combination of chitosan oligosaccharide nanoparticles and propolis, with CeraSeal serving as the control. The materials used for the preparation of nCOS‐P included chitosan oligosaccharide obtained from the Innovative Center of Excellence for Chitosan and Advanced Biomaterials at the Universitas Sumatera Utara, Medan, Indonesia. According to the Certificate of Analysis from the Research Center Laboratory of the same university, the chitosan powder had the following characteristics: it was an off‐white fine powder with a particle size finer than 100 mesh. Its chemical properties included a degree of deacetylation of 68.9%, a water solubility of 99.4%, a viscosity of 50.00 CPS, a moisture content of 4.00%, and an ash content of 1.10%. The Indonesian stingless bee propolis used in this study was sourced from Kebun Efi, located in North Sumatera, Indonesia.

### 2.2. Material Preparation Procedure

To prepare the propolis extract, 70 g of raw propolis was macerated in a 70% ethanol solution for 24 h. The macerated mixture was then frozen at –20°C for 10 h and subsequently centrifuged at 4500 rpm for 10 min. Following centrifugation, the supernatant was concentrated by evaporation at 40°C to obtain a pure propolis extract. The phytochemical profiles of the propolis extract can be found in our previous work [21, 23]. The propolis extract predominantly contained triterpenoids, including cycloartenol, amyrenone, and mangiferolic acid.

For the synthesis of nano propolis‐chitosan, the homogenization process began by mixing the propolis extract with chitosan oligosaccharide, gum arabic, and maltodextrin. The following is the formulation for the sealer: calcium silicate 41.26% (w/w), chitosan oligosaccharide 35.8% (w/w), propolis nanocapsules 2.94% (w/w), and zirconium oxide 20% (w/w). Meanwhile, gum arabic, maltodextrin, and propolis extract are used in the production of micro/nanoencapsulated propolis with a ratio of maltodextrin to gum arabic of 100:3 and a quantity of propolis extract amounting to 25% (w/w) of the total solids (maltodextrin and gum arabic).

Briefly, a total of 30 g of maltodextrin and 0.9 g of gum arabic were dissolved in 25 g of water until homogeneous, after which 10 g of concentrated propolis extract was added. The mixture was homogenized using an Omni International GLH 02 homogenizer (USA) at 4000 rpm for 60 s. The homogenization process was performed in two stages: initially, half of the weighed concentrated propolis extract was poured in and homogenized for 30 s, followed by the addition of the remaining extract and another 30 s of homogenization. The resulting homogeneous mixture of the encapsulant and concentrated propolis extract was then subjected to a preliminary freezing treatment using a Thermo Scientific Herafreeze Basic deep freezer (Germany) at −50°C overnight. Finally, the freeze‐drying process was carried out with a Christ Martin Alpha 1–2 LD Plus freeze dryer (Germany). The mixture was homogenized at 10,000 rpm for 5 min and then refrigerated at 4°C for 24 h to stabilize the formulation. Maltodextrin and gum arabic were added in the micro/nanoencapsulation of propolis using a freeze dryer as coating materials (wall material/protective carrier) that protect bioactive compounds in propolis that easily damage.

Maltodextrin provides solubility, forms a stable dry matrix, and reduces the hygroscopicity of the product, while gum arabic has good emulsification and film‐forming properties that help trap propolis particles within the matrix. The combination of the two improves the stability, solubility, rehydration, and shelf life of the micro/nanoencapsulation results while preventing the loss of volatile components or degradation of active compounds during the freeze‐drying process and storage. The stabilized mixture underwent microencapsulation through freeze‐drying, producing propolis‐chitosan microcapsules. These microcapsules were then milled using a high‐energy milling device to generate nanoparticles with an average size of 264.5 nm, as confirmed by a particle size analyzer (Horiba SZ‐100, Kyoto, Japan).

### 2.3. Material Characterization Procedure

For crystalline structure and elemental analysis, the nCOS‐P sealer was placed into an acrylic mold with a diameter of 10 mm and thickness of 2 mm and allowed to harden completely. The hardened sealer was then ground into a fine powder using a mortar and pestle. This powder was analyzed via X‐ray diffraction (XRD) to determine the material’s crystalline structure and identify its elemental composition based on diffraction intensity peaks.

XRD analysis was conducted using an X‐ray diffractometer, Bruker D8 Advance (Massachusetts, USA). The X‐ray source used was Cu‐Kα radiation with a wavelength of 1.5406 Å. The instrument operated at a voltage of 40 kV and a current of 30 mA. Detection was performed in θ–2θ scan mode, with a scanning angle range (2θ) from 5° to 80°. The scan speed was set at 2° per min. Samples were prepared in cylindrical form with a diameter of 10 mm and a thickness of 2 mm. Prior to the analysis, the samples were dried, ground into a fine powder, and placed in a sample holder. Crystallographic peak identification was carried out using the ICDD PDF‐2 database as the reference standard.

For surface morphology analysis, a similarly prepared nCOS‐P sample was examined using a scanning electron microscopy (SEM), equipped with energy‐dispersive X‐ray spectroscopy (EDS) (SEM‐EDS, Phenom XL G2, Thermo Fisher Scientific, Eindhoven, the Netherlands) to observe the surface characteristics and particle profiles. The operation was conducted in the high vacuum mode, using a secondary electron (SE) detector to observe surface topography. The accelerating voltage was set between 10 and 20 kV. Magnification ranged from 500× to 10,000×, with 5000× commonly used for endodontic materials. Sample preparation involved drying the sample, mounting it on an SEM stub, and coating it with a thin layer of gold using a sputter coater. The gold coating was applied at a current of ~30–60 mA for 60–120 s. The sample diameter was 10 mm.

EDS was also conducted to determine the atomic percentage (%At) of elements present in the material. EDS was conducted using both point scan and area scan detection modes. The energy spectrum range was set to be from 0 to 20 keV. Data acquisition was performed for 60–100 s per point. The resulting output provided the elemental composition expressed in both weight percentage (%wt) and %At.

### 2.4. Physical Properties Test Procedures

The setting time of the sealer was evaluated using a Vicat needle with a diameter of 1.13 mm and a length of 50 mm under a load of 300 g, following the ASTM C191‐08 standard. Measurements were taken at a room temperature of 23 ± 1°C and relative humidity of 50% ± 5%. The test was continued until the needle no longer left an indentation on the sample.

The flowability of the sealer was evaluated in accordance with ISO 6876:2012. Briefly, 0.05 mL of freshly mixed sealer was placed at the center of a glass plate measuring 40 mm× 40 mm with a thickness of 5 mm. At 180 ± 5 s from the start of mixing, a second glass plate and an additional load were applied to achieve a total mass of 120 g. The load was maintained for 10 min. After load removal, the maximum and minimum diameters of the compressed sealer disc were measured using a digital caliper (Vizbrite Electronic Digital Caliper), and the mean diameter was recorded as the flowability value.

For the film thickness test, 0.05 mL of freshly mixed sealer was placed at the center of a glass plate measuring 200 mm^2^ in area and 5 mm in thickness in accordance with ISO 6876:2012. A second glass plate of identical dimensions was carefully positioned over the material. The load application was initiated at 180 ± 5 s from the start of mixing. A vertical load of 150 N was applied for 10 min. The combined thickness of the two glass plates and the sealer film was measured using a pocket thickness gauge (Mitutoyo Corporation, Kawasaki, Japan). Film thickness was calculated by subtracting the combined thickness of the two glass plates without the sealer from the total thickness obtained after load application.

### 2.5. Data Analysis

For comparisons between two groups, the Kruskal–Wallis test is equivalent to the Mann–Whitney *U* test and was used as a non‐parametric approach to evaluate significant differences in setting time (min), flowability (mm), and film thickness (µm) between the nCOS‐P sealer and commercial bioceramic sealers.

## 3. Results

### 3.1. Particle Size

The particle size distribution of the chitosan‐propolis nanoparticles was measured using dynamic light scattering (DLS) with a HORIBA SZ‐100 analyzer at a scattering angle of 90° and a temperature of 25°C. The dispersion medium had a viscosity of 0.894 mPa·s. The particles exhibited a moderately polydisperse distribution with a Z‐average diameter of 327.0 nm and a polydispersity index (PDI) of 0.447, indicating moderate uniformity.

The mean particle size was 345.9 nm, with a median (D50) of 264.5 nm. The cumulative particle size distribution showed that 10%, 50%, 70%, and 90% of particles were smaller than 124.6 nm, 264.5 nm, 380.0 nm, and 664.0 nm, respectively. The mode of the distribution was 232.5 nm. These results confirm that the synthesized material falls within the nanoparticle size range, which is suitable for applications requiring enhanced penetration and surface interaction in endodontic sealing.

### 3.2. XRD

XRD analysis was performed to examine the diffraction patterns and crystalline composition of the nCOS‐P sealers and the commercial bioceramic sealer (CeraSeal). The XRD results showed that both sealers exhibited sharp diffraction peaks, indicating the presence of crystalline phases. The diffractogram of the nCOS‐P sealer (Figure 1A) displayed a semicrystalline pattern with nine distinct diffraction peaks, corresponding to the presence of calcium‐containing phases and zirconium‐based phases, including zirconium dioxide (ZrO_2_) (ICDD Number 00‐152‐8984) and silicon dioxide (SiO_2_) (ICDD Number 00‐412‐4076). In contrast, the diffractogram of CeraSeal (Figure 1B) revealed the presence of zirconium silicide (ZrSi_2_) (ICDD Number 00‐153‐2424), which was not detected in the nCOS‐P sealer.

Figure 12θ diffraction peaks of (A) nCOS‐P sealer and (B) commercial bioceramic sealer (CeraSeal). Morphological features: (C) nCOS‐P sealer and (D) commercial bioceramic sealer (CeraSeal) with SEM magnification ×5000. Microanalysis of particles and cementation phase of nCOS‐P sealer (E) and commercial bioceramic sealer (CeraSeal) (F) by EDS.

**Figure:**
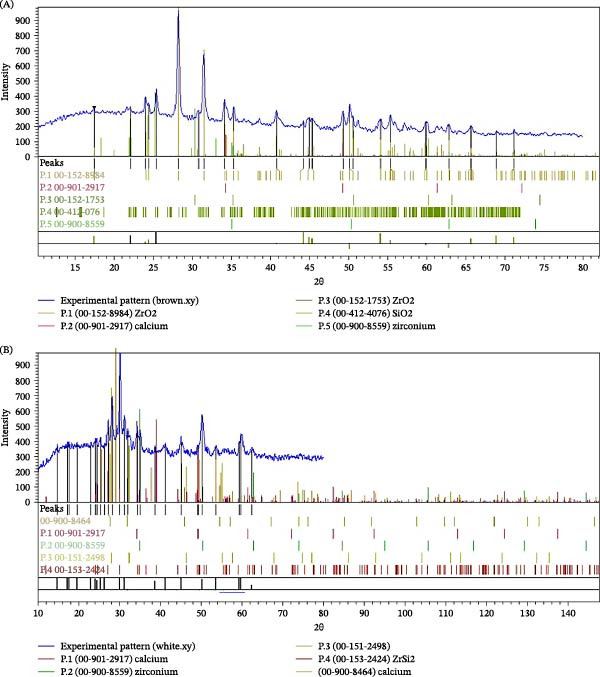

**Figure:**
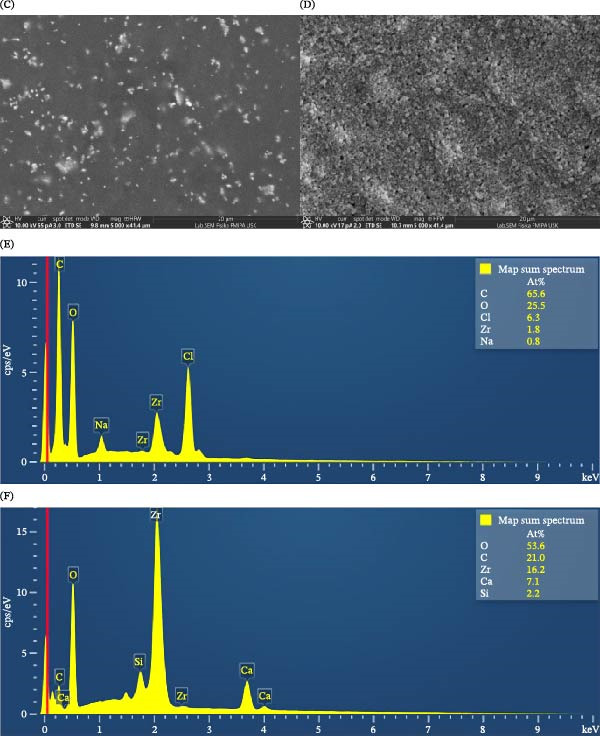

Table 1 shows that both sealers share nine similar 2θ diffraction peaks. The presence of the zirconium oxide phase in the nCOS‐P sealer contributes to its bioactivity, while the detection of silicon dioxide in CeraSeal suggests an association with silicate‐based components. These findings support the hypothesis that the nCOS‐P sealer exhibits a crystalline pattern comparable to that of CeraSeal, with notable differences in minor phases, such as the presence of ZrSi_2_ in CeraSeal, which is absent in nCOS‐P. Additionally, the presence of chitosan in nCOS‐P contributes to its semicrystalline structure, enhancing mechanical properties without compromising bioactivity. This is an essential characteristic for root canal‐filling applications.

**Table 1:** 2θ diffraction peaks of nCOS‐P sealer and commercial bioceramic sealer (CeraSeal).

2θ (°)	Sealer nCOS‐P	CeraSeal
17.42	✓	✓
24.02	✓	✓
25.38	✓	✓
28.20	✓	✓
34.12	✓	✓
40.78	✓	✓
44.24	✓	✓
59.96	✓	✓
62.84	✓	✓

### 3.3. SEM‐EDS

SEM‐EDS analysis was performed to evaluate the surface morphology, elemental distribution, and particle composition of both sealers. As shown in Figure 1C, the nCOS‐P sealer exhibits a nonuniform particle distribution, with certain areas appearing denser than others. In contrast, CeraSeal (Figure 1D) displays a more uniform and evenly dispersed particle distribution. These observations suggest that nCOS‐P has a more compact interparticle spacing, which may contribute to improved sealing ability within the root canal system.

The results of the EDS microanalysis (Figure 1E,F) reveal notable differences in the elemental composition between the two materials. Table 2 summarizes the major elements detected in both sealers. In the case of nCOS‐P, the presence of carbon and chlorine is attributed to the incorporation of propolis and chitosan, which not only enhance the viscosity but also contribute to the bioactivity of the material. Bioactivity is mainly associated with the calcium silicate phase when mixing nanochitosan with propolis (release of Ca(OH)_2_/alkalinity) and the organic components (cationic chitosan and propolis phenolic compounds) that influence viscosity, surface interactions, and antimicrobial activity. Conversely, the more homogeneous elemental distribution observed in CeraSeal is likely associated with its consistent formulation, which supports improved mechanical stability and handling characteristics during clinical applications.

**Table 2:** Percentage (At%) of elements in nCOS‐P and CeraSeal sealers based on EDS.

Elements	Sealer nCOS‐P (%)	CeraSeal (%)
Carbon (C)	65.6	21
Oxygen (O)	25.5	53.6
Chlorine (Cl)	6.3	–
Zirconium (Zr)	1.8	16.2
Sodium (Na)	0.8	–
Calcium (Ca)	–	7.1
Silicon (Si)	–	2.2

### 3.4. Setting Time

The setting time test was carried out using a Vicat needle in accordance with the ASTM C191−08 standard. As shown in Table 3, the nCOS‐P sealer exhibited a significantly faster setting time (1320 min) compared to the commercial sealer CeraSeal (3360 min) (*p*  < 0.05).

**Table 3:** Setting time of nCOS‐P and CeraSeal sealers.

Sealer	Median (min)	*p*‐Value
Sealer nCOS‐P	1320	<0.05
Sealer CeraSeal	3360	–

A shorter setting time is advantageous in clinical endodontic procedures, particularly those that require time efficiency, such as multirooted canal treatments or cases involving uncooperative patients. The rapid setting of nCOS‐P can help reduce chair time and minimize the risk of contamination during the procedure. This property is largely attributed to the water‐soluble nature of chitosan oligosaccharide, which enhances the viscosity and accelerates the hydration reaction, leading to faster hardening.

### 3.5. Flowability

Based on the results of the flowability test (Table 4), the nCOS‐P sealer exhibited a flowability of 19.05 ± 0.36 mm, which was significantly higher than that of CeraSeal (18.15 ± 0.26 mm) (*p*  < 0.05). This superior flowability indicates that nCOS‐P possesses lower viscosity, allowing it to spread more easily and penetrate narrow or irregular areas within the root canal system.

**Table 4:** Flowability of nCOS‐P and CeraSeal sealers.

Sealer	Flowability (mm)	*p*‐Value
Sealer nCOS‐P	19.05 ± 0.36	<0.05
Sealer CeraSeal	18.15 ± 0.26	–

### 3.6. Film Thickness

Film thickness testing revealed that nCOS‐P had a thickness of 180 ± 1.45 µm, which was lower than that of CeraSeal (260 ± 0.11 µm), as shown in Table 5. A lower film thickness is advantageous because it enables the sealer to better penetrate tight or constricted areas within the root canal system, ensuring closer adaptation to the canal walls.

**Table 5:** Film thickness of nCOS‐P and CeraSeal sealers.

Sealer	Film thickness (µm)	*p*‐Value
Sealer nCOS‐P	180 ± 1.45	<0.05
Sealer CeraSeal	260 ± 0.11	–

## 4. Discussion

The XRD test results indicate that the novel nCOS‐P sealer forms crystalline phases comprising calcium‐containing and zirconium‐based phases, including zirconium dioxide (ZrO_2_) and silicon dioxide (SiO_2_) of calcium (Ca) (ICDD Number 00‐901‐2917), zirconium (Zr) (ICDD Number 00‐900‐8559), zirconium dioxide (ZrO_2_) (ICDD Number 00‐152‐8984), and silicon dioxide (SiO_2_) (ICDD Number 00‐412‐4076). These phases differ from those found in the commercial bioceramic sealer CeraSeal, which contains calcium (Ca), zirconium (Zr), and zirconium silicide (ZrSi_2_) (ICDD Number 00‐153‐2424). Because XRD detects crystalline phases, even if there is a very small amount of zirconium oxide (ZrO_2_) present, a sharp peak may appear, even though the actual concentration of the element is very low. Neither propolis (a resinous bee product rich in polyphenols, flavonoids, and wax) nor chitosan (a type of polysaccharide) naturally contains zirconium. Zirconium oxide is deliberately added during the manufacture of sealers. The concentration of zirconium oxide added is 20% dry basis. The absence of the tricalcium silicate phase in nCOS‐P suggests that its composition is simpler than that of traditional calcium silicate‐based materials, such as Portland cement.

Khalill et al. [26] reported that tricalcium silicate typically appears at 2θ diffraction peaks between 32° and 34°, while the current study detected only a calcium silicate phase within that range. In addition, Wang et al. [27] showed that peaks in the 32°–34° 2θ range could also correspond to β‐dicalcium silicate. The combination of calcium silicate and zirconium oxide in the nCOS‐P formulation significantly contributes to its mechanical properties, which are further enhanced by chemical interactions between chitosan oligosaccharide and propolis.

Zirconium oxide in nCOS‐P also serves as a radiopacifier, offering excellent radiopacity without the risk of discoloration often associated with bismuth oxide [28]. This composition strengthens interparticle bonding, resulting in superior mechanical and bioactive performance compared to that of conventional bioceramic sealers. From a clinical perspective, radiopacity is required to verify sealer placement and to judge the quality of root canal obturation. In the present study, zirconium oxide was incorporated (20% w/w, dry basis) as the intended radiopacifier; however, ISO‐standard radiopacity measurements were not performed and are therefore a limitation of this report. Radiopacity testing (ISO 6876) will be included in the next phase of evaluation as part of our ongoing study.

SEM‐EDS analysis revealed a nonuniform elemental distribution in nCOS‐P when compared to that in CeraSeal. The higher carbon and chlorine signals observed in nCOS‐P are consistent with the presence of the propolis‐chitosan component, whereas CeraSeal exhibited a more mineral‐dominant elemental profile. These observations support the compositional distinction between the two materials but do not directly demonstrate improved sealing ability or biological performance. Specifically, some regions in nCOS‐P appeared denser than others, suggesting that the chitosan oligosaccharide acts as an adhesive, binding calcium silicate and zirconium oxide particles to form a cohesive matrix. Wang et al. [27] noted that chitosan helps wet the surfaces of calcium silicate particles, thereby enhancing interparticle adhesion. EDS shows a dominance of C and the presence of Cl, consistent with water‐soluble chitosan oligosaccharide in the hydrochloride form, indicating an organic polymer matrix at the particle interfaces. SEM shows more compact interparticle spacing, consistent with chitosan acting as a binder/wetting agent that wets calcium silicate surfaces and strengthens interpowder adhesion.

The particle size analysis showed that nCOS‐P had a smaller average particle size (2.678 ± 4.7 µm) compared to CeraSeal (2.132 ± 3.3 µm). This smaller size offers an advantage in penetrating the dentinal tubules, particularly in the narrower apical region. It should be noted that the nanoparticle size reported in Section 3.1 refers specifically to the chitosan‐propolis nanoparticles measured by DLS (nm scale), whereas the micron‐scale values discussed here represent the overall particle structure of the final sealer composite as observed by SEM. According to Giudice et al. [4], dentinal tubule diameters vary from 4.32 µm in the coronal third to 1.73 µm in the apical third, indicating that the finer particles of nCOS‐P may improve sealing efficacy in these challenging regions. Additionally, the high carbon (C) and chlorine (Cl) content in nCOS‐P, derived from propolis and chitosan, contributes to its high viscosity and may accelerate the hydration reaction. Propolis, rich in phytochemicals, supports the material’s bioactivity, while chitosan functions as a biocompatible thickening agent [29].

Setting time is a critical parameter in the clinical application of sealers. In this study, the novel nCOS‐P sealer exhibited a significantly shorter setting time (1320 min) compared to CeraSeal (3360 min). This rapid setting is attributed to the water‐soluble nature of chitosan oligosaccharide, which increases viscosity and accelerates hydration gel formation [30, 31]. On the other hand, CeraSeal’s slower setting time is due to its premixed formulation, which requires activation by tissue fluids to initiate hardening. However, this finding should be interpreted cautiously because premixed calcium silicate‐based sealers are moisture‐activated and may show delayed setting under relatively dry laboratory conditions. Therefore, the present study reflects a comparative laboratory measurement under standardized room conditions rather than a direct estimate of clinical setting behavior. Loushine et al. [32] found that premixed bioceramic sealers often show delayed setting in dry root canals, consistent with the observations in this study. A faster setting time provides clinical advantages in procedures requiring time efficiency, such as complex or multiroot canal fillings. However, in certain clinical scenarios, a slower setting time may be beneficial by offering an extended working time for more precise application.

Flowability testing revealed that the novel nCOS‐P sealer demonstrated a higher flowability value (19.05 mm) than CeraSeal (18.15 mm), both of which met the ISO 6876/2012 standard (>17 mm). This higher flowability reflects the lower viscosity of nCOS‐P, enabling the material to reach narrower canal regions and improve tubule penetration. While high flowability enhances sealing ability, it may also increase the risk of leakage if it is not carefully managed during clinical application. In terms of film thickness, nCOS‐P showed a higher value (120 µm) than that of CeraSeal, exceeding the ISO 6876/2012 standard (≤50 µm). Although increased film thickness could potentially affect the density and compactness of the filling, the presence of chitosan oligosaccharide in nCOS‐P improves its washout resistance in the presence of tissue fluids, thereby enhancing material stability [33].

Clinical handling and predictability are strongly influenced by canal moisture. Conventional sealers are often placed after drying protocols and may not require moisture to set, whereas premixed calcium silicate‐based bioceramic sealers are hydration‐driven and therefore moisture‐dependent; insufficient moisture can delay setting time in laboratory conditions, which is clinically relevant in very dry canals [15]. A recent systematic review of comparative clinical studies reported similar tooth survival, treatment outcomes, postoperative pain, and periapical extrusion between premixed bioceramic sealers and standard sealers [34]. In addition, a 3‐year clinical outcome study using CeraSeal with a single‐cone technique reported favorable survival and success, supporting the clinical viability of premixed bioceramic sealers in diverse conditions [35]. These clinical data support the translational relevance of evaluating the physicochemical properties of nCOS‐P against a premixed bioceramic comparator.

Overall, this study demonstrates that the novel, natural‐based nCOS‐P sealer exhibits promising physical and bioactive properties when compared to the commercial bioceramic sealer, CeraSeal. It offers clear advantages in terms of setting time, flowability, and particle distribution. However, certain limitations, such as exceeding the standard film thickness, highlight the need for further optimization. These findings represent a valuable contribution to the development of naturally derived endodontic materials. Nevertheless, additional studies are required to assess the biological performance, such as radiopacity, solubility, dimensional stability, washout resistance, sealing ability, and clinical efficacy, of nCOS‐P under conditions that more closely replicate the oral environment.

## 5. Conclusions

In conclusion, the novel nCOS‐P sealer shows potential as a natural‐based alternative to commercial bioceramic sealers. Compared with CeraSeal, it demonstrated a faster setting time and higher flowability. However, its film thickness exceeded ISO requirements, indicating that further formulation optimization is needed. Additional studies are required to evaluate its radiopacity, physicochemical stability, biological performance, and long‐term clinical efficacy.

## Funding

This research was funded by the Universitas Sumatera Utara General Research Grant.

## Disclosure

The funders had no role in the study design, data collection, analysis, or interpretation, in the writing of the report, or in the decision to submit the article for publication. The propolis was supplied by Kebun Efi.

## Ethics Statement

No ethical approval was required for this study.

## Conflicts of Interest

Felix Zulhendri is affiliated with Kebun Efi, which supplied the propolis for this work. The other authors declare no conflicts of interest.

## Data Availability

The data that support the findings of this study are available from the corresponding author upon reasonable request.
